# Preparation and functional evaluation of collagen oligopeptide-rich hydrolysate from fish skin with the serine collagenolytic protease from *Pseudoalteromonas* sp. SM9913

**DOI:** 10.1038/s41598-017-15971-9

**Published:** 2017-11-16

**Authors:** Xiu-Lan Chen, Ming Peng, Jing Li, Bai-Lu Tang, Xuan Shao, Fang Zhao, Chang Liu, Xi-Ying Zhang, Ping-Yi Li, Mei Shi, Yu-Zhong Zhang, Xiao-Yan Song

**Affiliations:** 1 State Key Laboratory of Microbial Technology, Marine Biotechnology Research Center, Institute of Marine Science and Technology, Shandong University, Jinan, 250100 China; 2 Laboratory for Marine Biology and Biotechnology, Qingdao National Laboratory for Marine Science and Technology, Qingdao, 266237 China

## Abstract

Although several serine collagenolytic proteases from bacteria were reported, none has been used to prepare bioactive collagen peptides. MCP-01 is the most abundant extracellular protease of deep-sea *Pseudoalteromonas* sp. SM9913 and is a serine collagenolytic protease with high efficiency on fish collagen hydrolysis. Here, we set up a pilot scale process to ferment SM9913 for extracellular protease production. With SM9913 extracellular protease as a tool, a process to prepare collagen oligopeptide-rich hydrolysate from codfish skin was set up, which was further scaled up to pilot (100 L) and plant (2000 L) levels with yields >66%. The hydrolysates from laboratory-, pilot- and plant-scales had quite similar quality, containing ~95% peptides with molecular weights lower than 3000 Da and approximately 60% lower than 1000 Da, in which collagen oilgopeptides account for approximately 95%. Bioactivity analyses showed that the hydrolysate had moisture-retention ability, antioxidant activity, and promoting effect on cell viability of human dermal fibroblasts. Safety evaluation showed that the hydrolysate was nontoxic and nonirritating to skin. Therefore, SM9913 extracellular protease is a good enzyme to prepare bioactive oligopeptides from fish skin. The results also suggest that the collagen oligopeptides-rich hydrolysate may have potentials in biomedical, functional food, pharmaceutical and cosmetic industries.

## Introduction

Bioactive oligopeptides, which contain only 2–10 amino acid residues with molecular weights of less than 1,500 Da, have received much attention due to their numerous potential physiological functions, including angiotensin-I-converting enzyme (ACE) inhibitory activity^1^, antimicrobial activity^2^, antioxidative^3^ and immunomodulatory^4^ properties etc. In addition, bioactive oligopeptides possess multitudinous properties, such as oil absorption ability, protein solubility, water holding ability, foaming ability, gelling activity and emulsification capacity^5^. Studies also showed that small peptides containing 2–5 amino acid residues are more easily absorbed than free amino acids with the aid of peptide transport systems^6^. For these reasons, bioactive oligopeptides have been widely used in functional food^7^, pharmaceutical^8^ and cosmetic industries^9^. Traditionally, bioactive peptides can be produced by four methods including transgene, recombination, synthesis and enzymatic hydrolysis^10^. Enzymatic hydrolysis is a preferred method because it is more suitable for large-scale applications and more environmentally friendly than the other methods^10^. In recent years, bioactive collagen oligopeptides are quite popular in many countries. They are used as functional foods among the general public with various health problems such as gastrointestinal disorders^11^. Many brands of commercially functional foods containing oligopeptides prepared from fish proteins, such as Amizate^®^, Stabilium^®^ 200 and Nutripeptin^®^ are reported to have health-promoting effect^12,13^. Collagen peptides are also used as antioxidant agents, cryoprotective agents and moisturizing ingredients in cosmetic industries for their antioxidant activity, cryoprotective ability and moisture-retention ability^14^.

Fish skin is usually a by-product of fish processing industry. Every year, fish processing industry produces a considerable number of by-products accounting for ~70–85% of the total weight of catch, 30% of which is in the form of bones and skins. Because it is rich in collagen, fish skin is a good material for preparing bioactive collagen oligopeptides to improve its additional value. Protein hydrolysates have been reported to be prepared from some fish skins, such as *Lutjanus vitta* ^15^, and *Priacanthus macracanthus* ^16^. It has been showed that hydrolysates from fish skin usually contain multitudinous bioactive peptides, such as antimicrobial peptides^2^, immunomodulatory peptides^4^, antioxidative peptides^3^, and ACE inhibitory peptides^1^. These studies suggest that proteins in fish skin contain various bioactive sequences that can be released by enzyme hydrolysis.

Because low-molecular-weight oligopeptides containing 2–5 amino acid residues are easy to be absorbed, it is ideal for a protein hydrolysate to have high content of low-molecular-weight oligopeptides with strong bioactivity. To achieve this, choosing a suitable protease is essential for the production of oligopeptides-rich hydrolysate. Serine collagenolytic proteases have multiple cleaveage sites on collagen and can efficiently hydrolyze collagen^17,18^. Therefore, they may be good enzymes for collagen peptide production. However, though many kinds of proteases have been used to prepare collagen peptide hydrolysate, including plant proteases (papain, bromelain)^19^, bacterial proteases (alcalase, neutrase)^20^, animal proteases (trypsin, collagenase)^21^ and protamex^22^, there has been no report to prepare collagen peptide hydrolysate with a serine collagenolytic protease as a tool.

*Pseudoalteromonas* sp. SM9913 (hereafter SM9913) is a protease-secreting strain isolated from deep-sea sediment^23^. The most abundant extracellular protease of this strain is a serine collagenolytic protease MCP-01, which can efficiently hydrolyze collagen from fish skin into peptides^18^. In this study, we aimed to prepare bioactive and collagen oligopeptides-rich hydrolysate from fish skin with the extracellular protease from SM9913. We firstly optimized the fermentation conditions of SM9913 for extracellular protease production, and conducted pilot-scale fermentation in a 200 L fermentor. Then a process to prepare collagen oligopeptide-rich hydrolysate from fish skin with the extracellular protease from SM9913 was set up and was scaled up to pilot and plant scales. Furthermore, the bioactivities of the plant-scale hydrolysate, including moisture-absorption and retention abilities, antioxidant activity and its promotion effect on human cell proliferation, as well as its security evaluation, were investigated to assess its potentials in biotechnology and industry.

## Results and Discussion

### Fermentation optimization and pilot-scale production of SM9913 extracellular protease

**Figure 1:**
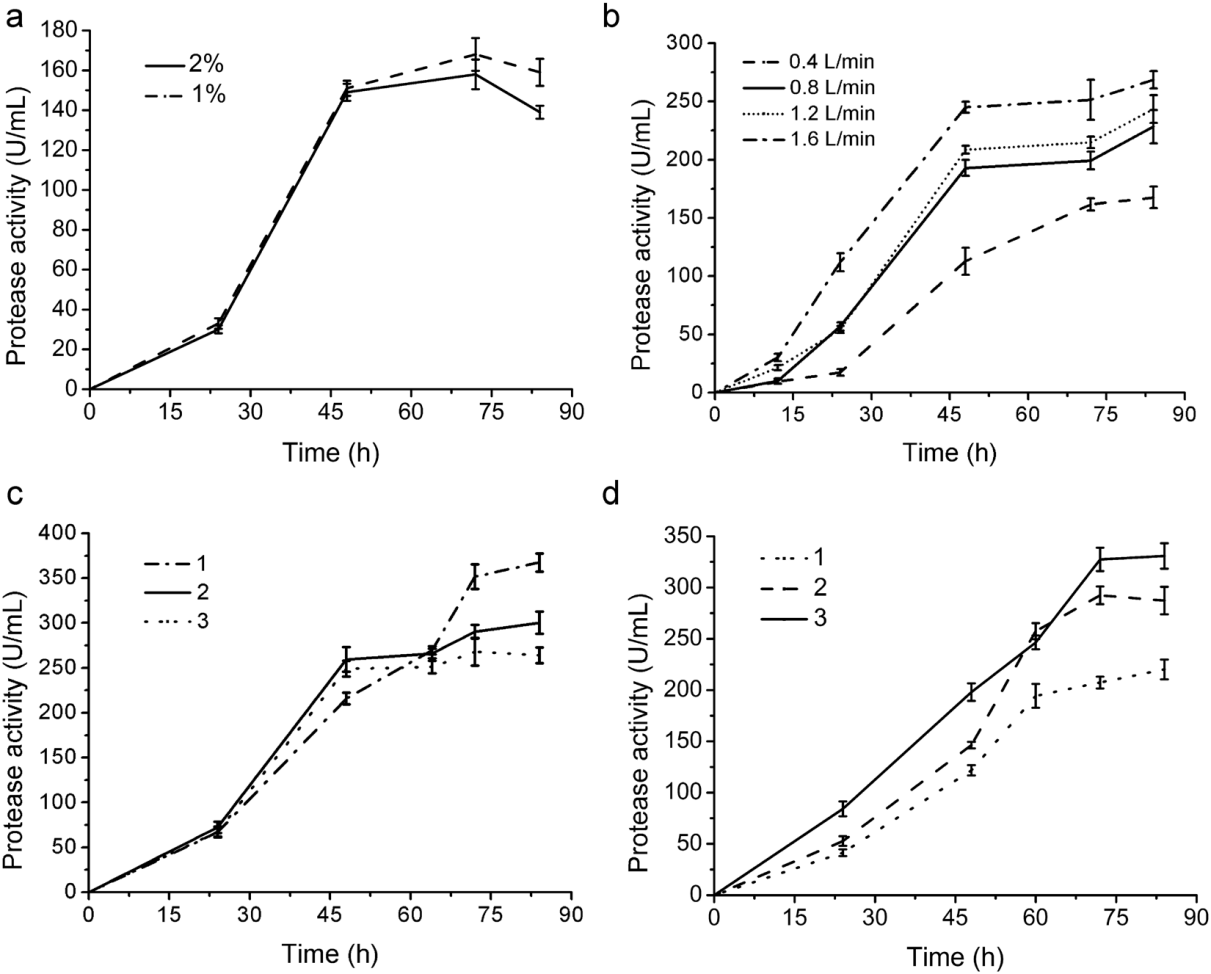
Effects of key factors on extracellular protease production of SM9913. (**a**) Effect of inoculation amount in shake flask fermentation. (**b**) Effect of gas flow in a 3 L fermentor. (**c**) Effect of stirring speed in a 3 L fermentor. Line 1, fermentation with a stirring speed of 400 rpm in the first 48 h and 350 rpm in the remaining time; line 2, fermentation with a stirring speed of 500 rpm in the first 48 h and 450 rpm in the remaining time; line 3, fermentation with a stirring speed of 500 rpm in the whole process. (**d**) Protease activity in the broth of SM9913 fermentation in a 200 L fermentor. Line 1, fermentation with a gas flow of 2 m^3^/h and a stirring speed of 90 rpm; line 2, fermentation with a gas flow of 4 m^3^/h and a stirring speed of 80 rpm; line 3, fermentation with a gas flow of 6 m^3^/h and a stirring speed of 100 rpm.

SM9913 is a cold-adapted strain from deep-sea sediment and can secrete a large amount of protease^23^. A flask fermentation process for SM9913 extracellular protease production was previously set up, in which SM9913 was cultured at 200 rpm, 12 °C for 72 h in a medium as described in Methods^23^. Based on this process, we further conducted small- and pilot-scale fermentation for SM9913 extracellular protease production. We first studied the influence of inoculation amount on the production of SM9913 protease in shake flask fermentation. As shown in Fig. 1a, when SM9913 was cultured at 15 °C for 84 h, the protease activity in the broth with 1% inoculation amount was a little higher than that with 2% inoculation amount, which reached 168 ± 8.21 U/mL. Hence, 1% inoculation amount was adopted in the following small- and pilot-scale fermentation.

The effects of aeration rate and stirring speed were investigated on the production of SM9913 protease in a 3 L fermentor. The protease production of SM9913 rose with the increase of aeration rate, reaching the maximum of 268 ± 7.33 U/mL at the aeration rate of 1.6 L/min (Fig. 1b). The stirring speed also affected SM9913 protease production. When the stirring speed in the fermentation broth was 400 rpm in the first 48 h and 350 rpm in the remaining time of fermentation, the protease production of SM9913 reached the maximum of 367 ± 10.24 U/mL (Fig. 1c).

Based on the determined parameters in small-scale fermentation, we further conducted pilot-scale fermentation of SM9913 in a 200 L fermentor. As in the small-scale fermentation, aeration rate and stirring speed that were regarded as two significant factors for the production of SM9913 protease were optimized in pilot-scale fermentation. As shown in Fig. 1d, when the aeration rate was 2 m^3^/h with the stirring speed of 90 rpm, the protease activity in the broth reached 220 ± 9.65 U/mL, which reached 287 ± 13.37 U/mL under the aeration rate of 4 m^3^/h with the stirring speed of 80 rpm, and 330 ± 12.39 U/mL under the aeration rate of 6 m^3^/h with the stirring speed of 100 rpm. Through optimization, we determined the pilot-scale fermentation process of SM9913 in a 200 L fermentor. With 1% inoculation amount and 70% loading volume, SM9913 was cultured at 15 °C for 72 h in a 200 L fermentor. The stirring speed was kept at 100 rpm at the first 48 h and then adjusted to 90 rpm for the remaining time of fermentation. The aeration rate was kept at 6 m^3^/h during the whole fermentation process. With this process, the protease activity in the fermentation broth of SM9913 reached ~340 U/mL, about two times of that in shake flask fermentation.

Although some proteases secreted by marine sedimentary bacteria have been characterized, reports on the pilot- and plant-scale production of these proteases are still rather less. Shao *et al*. optimized the culture conditions for the production of myroilysin from strain *Myroides profundi* D25 by using single factor experiments, which resulted in a significant improvement in myroilysin production in pilot-scale fermentation^24^. In this study, we successfully set up a pilot-scale fermentation process for the extracellular protease production of SM9913, laying a good foundation for the industrial application of SM9913 protease.

### Preparation of fish skin hydrolysate with SM9913 extracellular protease on laboratory, pilot and plant scales

**Figure 2:**
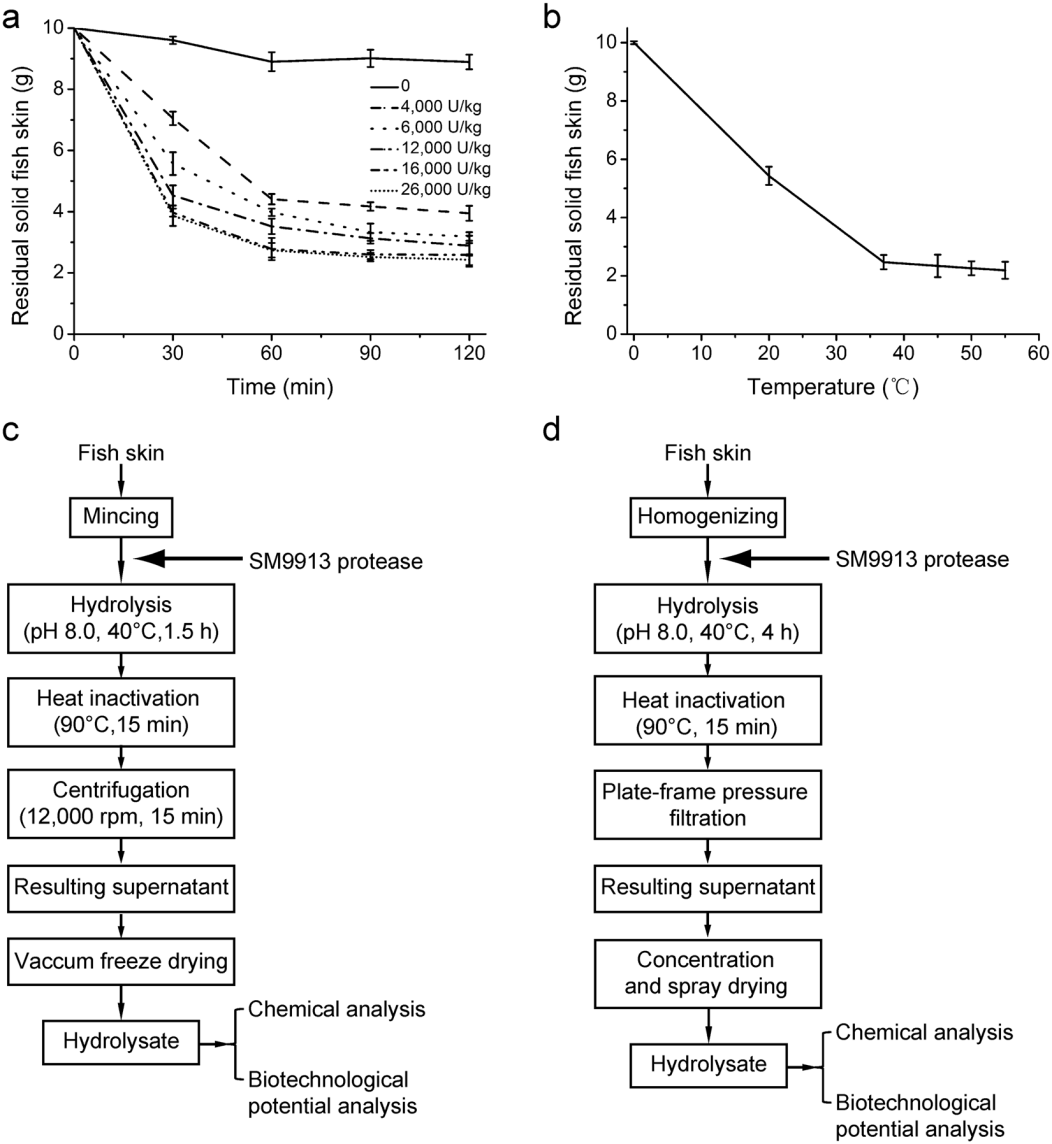
Effects of E/S, hydrolysis time and temperature on fish skin hydrolysis. (**a**) Effects of E/S and hydrolysis time. (**b**) Effect of hydrolysis temperature. (**c**) Flow sheet for the preparation of fish skin hydrolysate with SM9913 protease on laboratory scale. (**d**) Flow sheet for the preparation of fish skin hydrolysate with SM9913 protease at pilot and plant scales.

It has been shown that SM9913 secretes two extracellular proteases, MCP-01 and MCP-02, and the fermentation broth of SM9913 contained a predominant amount of MCP-01 and a very small amount of MCP-02^23^. MCP-01 is a serine collagenolytic protease of the S8 family with various cleavage sites on collagen and has higher activity to fish collagen (10434 U/mg) than to bovine tpye I collagen (1608 U/mg)^18,25^. In contrast, MCP-02 is a metalloprotease of the M4 family^23,26^ with only a much smaller activity toward collagen (207 U/mg to bovine tpye I collagen, unpublished data). These data suggest that the extracellular protease of SM9913 may be suitable to hydrolyze fish skin to prepare bioactive collagen peptides. Therefore, we studied the process to prepare fish skin hydrolysate with the extracellular protease of SM9913 from pilot-scale fermentation broth. To set up the process, three parameters, including enzyme-substrate ratio (E/S), hydrolysis temperature and hydrolysis time, were optimized by single factor experiments with the residual amount of solid fish skin substrate as the response. The results showed that fish skin hydrolysis reached the highest degree when E/S was higher than 16,000 U/kg, hydrolysis time was more than 1 h (Fig. 2a), and hydrolysis temperature was 40–55 °C (Fig. 2b). Based on these results, the E/S, hydrolysis temperature and hydrolysis time were determined to be 20,000 U/kg, 40 °C and 1.5 h, respectively. With these optimized parameters, a laboratory-scale process to produce fish skin hydrolysate with the extracellular protease of SM9913 was set up (Fig. 2c). With this process, ~74% of fish skin was degraded and the yield of the hydrolysate was 71.24% (d/d) (Table 1), indicating that it was an efficient process for fish skin hydrolysis. Due to its high efficiency, this process was further scaled up to pilot and plant levels (Fig. 2d). In the pilot- and plant-scale processes, E/S and hydrolysis temperature were the same as those in the laboratory-scale process, and hydrolysis time was extended to 4.0 h. The yields of the hydrolysates at pilot and plant scales were 67.93% and 66.58% (d/d), respectively (Table 1), indicating that a process for fish skin hydrolysate production at pilot and plant scales was successfully set up with SM9913 extracellular protease as a tool. It is worth to mention that, the other extracellular proteases secreted by SM9913, despite in a small amount, may have synergistic effect with MCP-01 in fish skin hydrolysis.

**Table 1:** Contents and recoveries of free amino acids and peptides in the fish skin hydrolysates prepared at different scales.

Hydrolysate	Content		Yield	
	Free amino acid (%)	Peptides (%)	d/w^*a*^ (%)	d/d^*b*^ (%)
Laboratory scale	3.48 ± 0.11	96.52 ± 0.11	12.49	71.24
Pilot scale	6.40 ± 0.57	93.60 ± 0.57	11.89	67.93
Plant scale	5.02 ± 0.76	94.97 ± 0.76	11.64	66.58

^a^Dry weight of hydrolysate powder/wet weight of solid fish skin substrate.

^b^Dry weight of hydrolysate powder/dry weight of solid fish skin substrate.

### Analysis of amino acids and peptides in the hydrolysates

**Figure 3:**
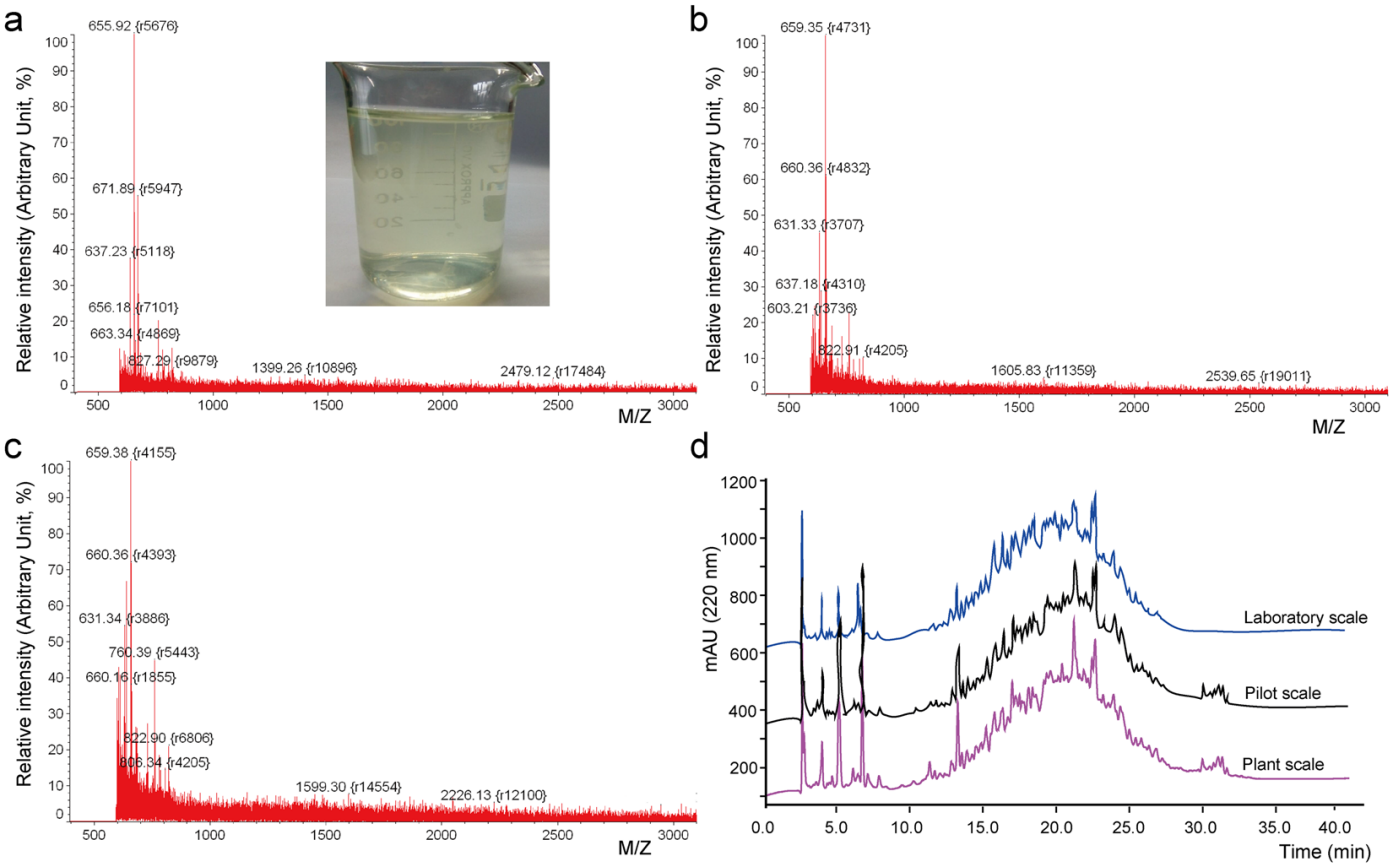
Characteristic analyses of fish skin hydrolysates prepared at laboratory, pilot and plant scales. (**a**,**b**,**c**) MALDI-TOF-MS analysis of the molecular weight distribution of peptides in the hydrolysate from laboratory (**a**), pilot (**b**) and plant (**c**) scales. Inset, solution of the hydrolysate (20 mg/mL). (**d**) RP-HPLC analysis of the peptide spectra of the hydrolysates from different scales.

**Figure 4:**
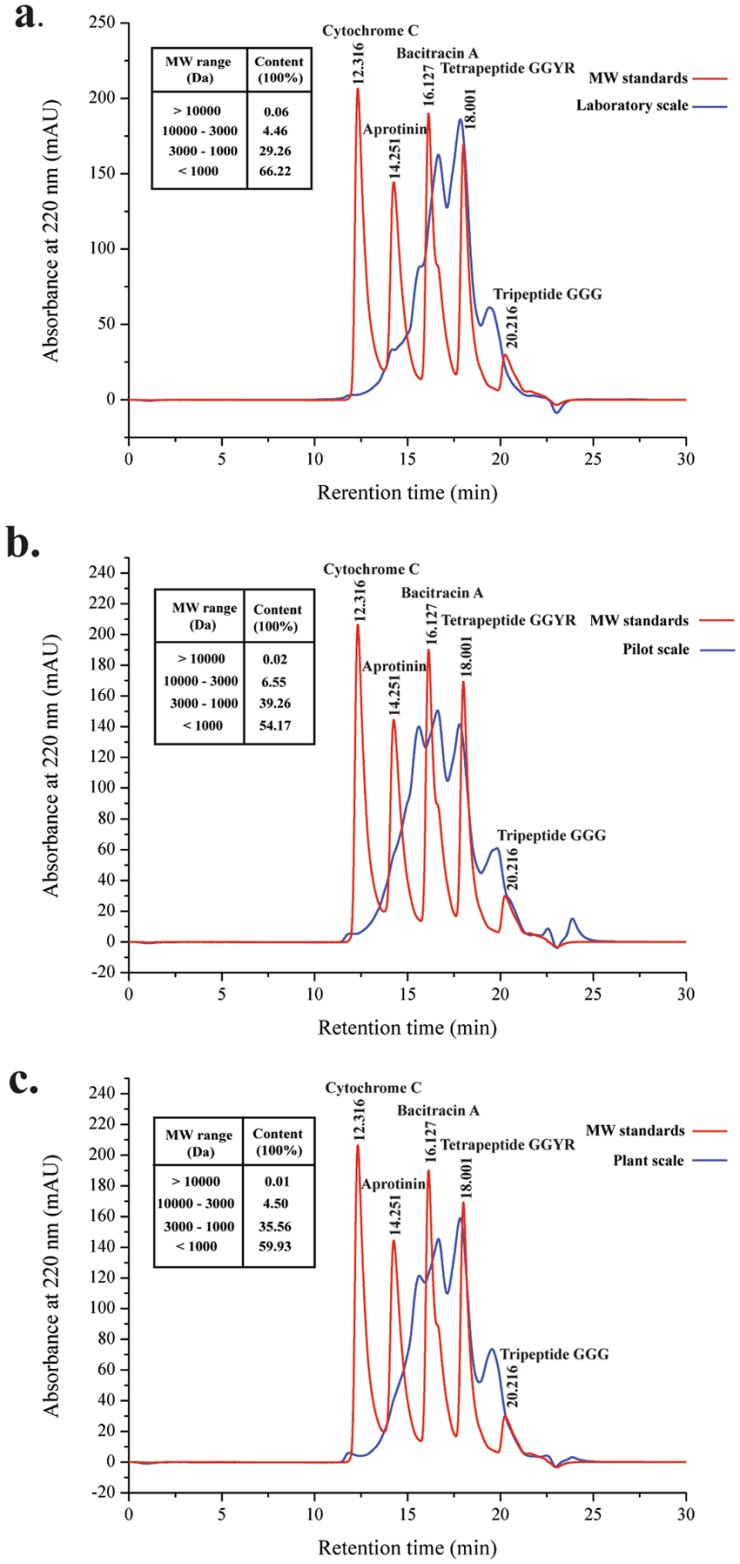
Size exclusion chromatography analysis of the molecular mass distribution of fish skin hydrolysates at laboratory (**a**), pilot (**b**) and plant (**c**) scales. Values above the peaks indicate the retention times of the standards. The retention times for the standards Tripeptide GGG (Mr 189), tetrapeptide GGYR (Mr 451), bacitracin (Mr 1422), aprotinin (Mr 6511) and cytochrome C (Mr 12400) are 20.216 min, 18.001 min, 16.127 min, 14.251 min and 12.316 min, respectively. (Inset) Contents of peptides in different molecular mass ranges in the hydrolysates at laboratory, pilot and plant scales.

The dried powder of the fish skin hydrolysates we prepared was completely water soluble and the solution was clear and non-bitter, with a pale yellow color (Fig. 3a inset). It has been reported that the biological activities and physicochemical properties of protein hydrolysate rely on the molecular weight distribution of peptides and amino acid composition of the hydrolysate, which are the most significant factors in the preparation of bioactive peptides with the desirable biological activities^27^. Therefore, prior to investigate the biological activities of the fish skin hydrolysate we prepared, the contents of free amino acids and peptides, amino acid composition and molecular weight distribution of peptides in the hydrolysates from different productive levels were analyzed. As shown in Figs 3 and 4 and Tables 1 and 2, these parameters of the hydrolysates were quite similar at different scales, indicating that the hydrolysates prepared at laboratory, pilot and plant scales remain fairly consistent quality. The peptide contents were higher than 93% in the hydrolysates (Table 1), showing that they are peptide-rich hydrolysates. The molecular mass distribution and peptide spectra of the hydrolysates at laboratory, pilot and plant scales were analyzed with MALDI-TOF-MS and RP-HPLC, respectively (Figs 3 and 4). MS spectra showed that the molecular masses of the peptides in the hydrolysates mainly ranged from 600 to 1,000 Da (Fig. 3a–c). The peptide spectra of the three hydrolysates were quite similar (Fig. 3d), indicating that they have similar peptide composition. We further analyzed the hydrolysates with size exclusion chromatography (Fig. 4). Approximately 95% of the peptides in the hydrolysates from different scales had a molecular mass lower than 3 kDa, in which the amount of peptides with a molecular mass lower than 1 kDa was around 60%, indicating that the hydrolysates were rich in oligopeptides.

**Table 2:** Composition and contents of amino acids in the fish skin, collagen from fish skin and the hydrolysates prepared at different scales.

Amino acid	Fish skin (g/100 g)	Collagens from fish skin (g/100 g)	Laboratory scale (g/100 g)	Pilot scale (g/100 g)	Plant scale (g/100 g)
Ala	5.83 ± 1.55	8.29 ± 1.43	6.56 ± 0.63	7.25 ± 2.06	9.07 ± 1.21
Arg	9.57 ± 0.24	7.58 ± 0.31	8.72 ± 0.03	7.77 ± 0.92	8.96 ± 0.11
Asn^*a*^	—	—	—	—	—
Asp	7.38 ± 0.31	6.03 ± 0.18	5.54 ± 0.19	5.83 ± 0.33	5.53 ± 0.19
Cys^*b*^	—	—	—	—	—
Gln^*a*^	—	—	—	—	—
Glu	12.33 ± 0.44	10.67 ± 0.24	9.89 ± 0.07	10.31 ± 0.41	10.10 ± 0.21
Gly	14.82 ± 0.79	22.30 ± 0.34	22.85 ± 0.18	21.98 ± 0.38	21.73 ± 0.33
His	1.27 ± 0.10	0.93 ± 0.03	0.91 ± 0.02	0.94 ± 0.07	0.79 ± 0.03
Ile^*^	1.73 ± 0.13	1.03 ± 0.04	0.93 ± 0.02	1.05 ± 0.01	0.92 ± 0.04
Leu^*^	3.62 ± 0.22	2.19 ± 0.04	1.86 ± 0.02	2.10 ± 0.05	2.06 ± 0.06
Lys^*^	4.59 ± 0.33	3.07 ± 0.09	3.01 ± 0.06	3.21 ± 0.27	3.03 ± 0.08
Met^*^	1.16 ± 0.06	0.72 ± 0.09	0.77 ± 0.04	0.93 ± 0.19	0.54 ± 0.04
Phe^*^	4.93 ± 0.26	6.13 ± 0.18	6.46 ± 0.27	6.14 ± 0.21	5.75 ± 0.26
Pro	6.68 ± 0.17	8.79 ± 0.03	8.53 ± 0.07	8.43 ± 0.33	7.92 ± 0.09
Ser	4.82 ± 0.23	5.01 ± 0.14	4.85 ± 0.08	4.82 ± 0.33	4.47 ± 0.14
Thr^*^	3.81 ± 0.20	3.03 ± 0.08	2.61 ± 0.07	2.74 ± 0.04	2.82 ± 0.09
Trp^*,*a*^	—	—	—	—	—
Tyr	3.64 ± 0.10	1.70 ± 0.95	2.20 ± 0.10	2.53 ± 0.16	2.99 ± 0.35
Val^*^	2.53 ± 0.15	1.62 ± 0.04	1.44 ± 0.03	1.52 ± 0.17	1.41 ± 0.01
Hypro	5.49 ± 0.11	8.33 ± 0.28	8.01 ± 0.24	7.80 ± 0.50	8.09 ± 0.49
Hylys	0.38 ± 0.04	0.53 ± 0.02	0.48 ± 0.02	0.53 ± 0.02	0.59 ± 0.03
Total	94.58 ± 5.43	97.95 ± 4.51	95.62 ± 2.14	95.88 ± 6.45	96.77 ± 3.76

*Human-essential amino acids.

^a^Not detectable because they were destroyed in the process of acid hydrolysis.

^b^Not detectable because of its low content in collagen.

—Not detectable.

**Figure 5:**
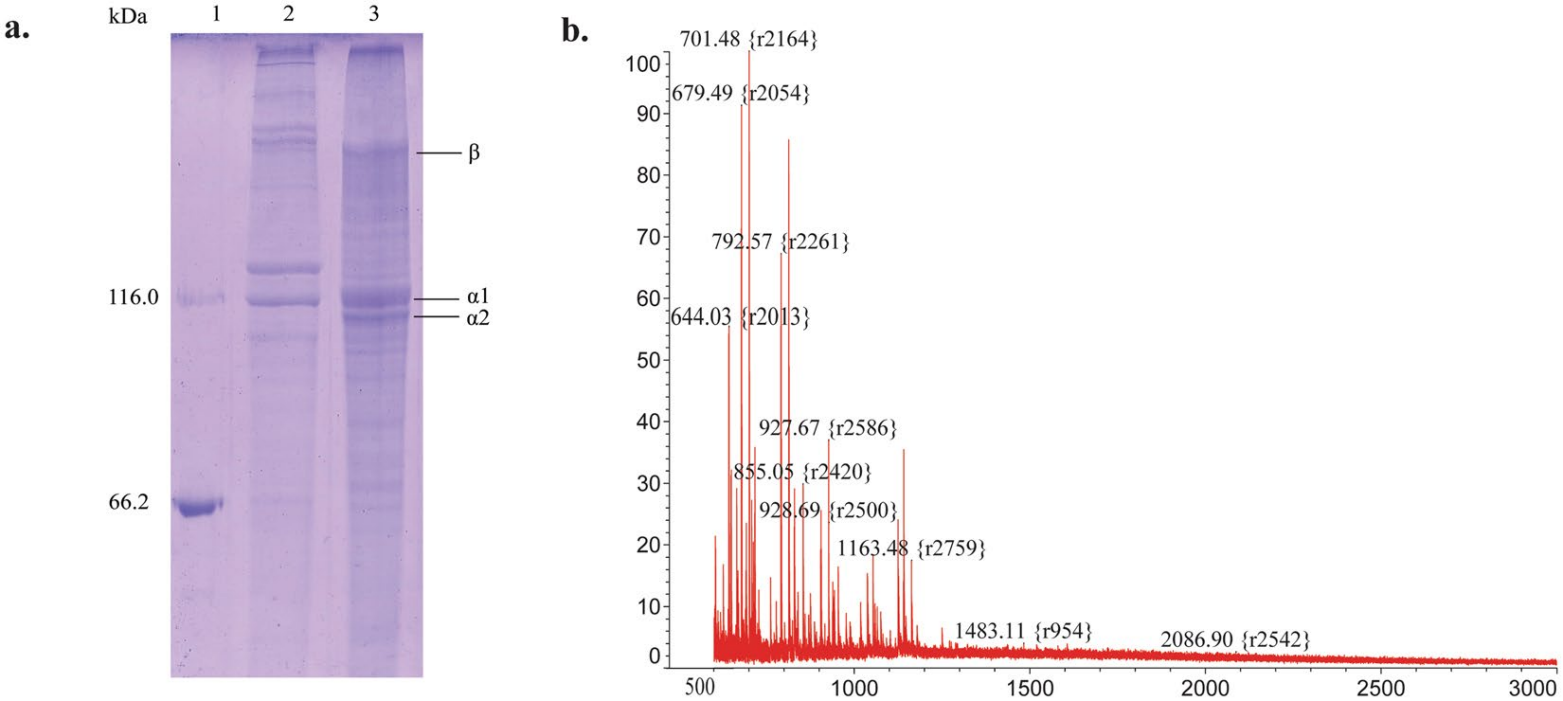
(**a**) SDS-PAGE analysis of the collagen extracted from the codfish skin. Lane 1, standard molecular mass marker; Lane 2, type I collagen from bovine Achilles tendon; lane 3, collagen from codfish skin. (**b**) MALDI-TOF-MS analysis of the hydrolysate from the codfish skin collagen digested by the extracellular protease of SM9913 at 60 °C for 5 h.

Hydroxylproline and hydrolysine are typical amino acids of collagen and rare in other proteins. Our results showed that the hydroxylproline content in the hydrolysates were approximately 8%, and the hydrolysine content is 0.53% (Table 2). This indicates that the protease MCP-01 from SM9913 can digest the collagen in codfish skin into oligopeptides. To support this conclusion, we further extracted collagen from the codfish skin and digested it by the extracellular protease from SM9913. As shown in Fig. 5a, the SDS-PAGE patterns of the collagen from codfish skin and type I collagen from bovine Achilles tendon were similar, but the molecular weights of the subunits of the two kinds of collagen had difference. This difference is likely due to the different sources of the two kinds of collagen, because previous studies have shown that the molecular weights of the subunits of collagen from different sources may be different^28–30^. After the codfish collagen was digested, most of the peptides released from the collagen were oligopeptides lower than 1 kDa (Fig. 5b). To further evaluate the content of collagen peptides in the hydrolysates, the amino acid composition of the codfish skin and of the collagen extracted from the codfish skin were analyzed. The result showed that the hydroxylproline content in the collagen from codfish skin was 8.33%. Therefore, based on hydroxylproline content, the hydrolysates should contain ~95% collagen peptides.

Taken together, these data indicate that the hydrolysates produced from fish skin with the extracellular protease of SM9913 at laboratory, pilot and plant scales have consistent quality, containing approximately 95% oligopeptides lower than 3 kDa and approximately 60% oligopeptides lower than 1 kDa, in which the content of collagen peptides is ~95%.

Studies have showed that hydrolysates from fish skin usually contain multitudinous bioactive peptides, such as antimicrobial peptides^2^, immunomodulatory peptides^4^, antioxidative peptides^3^, and ACE inhibitory peptides^1^, and therefore, are widely applied in functional food, pharmaceutical and cosmetic industries. The hydrolysates we prepared from fish skin mainly contain collagen oligopeptides, which may be rich in bioactive peptides. Thus, we further investigated the bioactivities of the plant-scale hydrolysate we prepared from fish skin.

### Moisture-absorption and retention abilities of the hydrolysate

**Figure 6:**
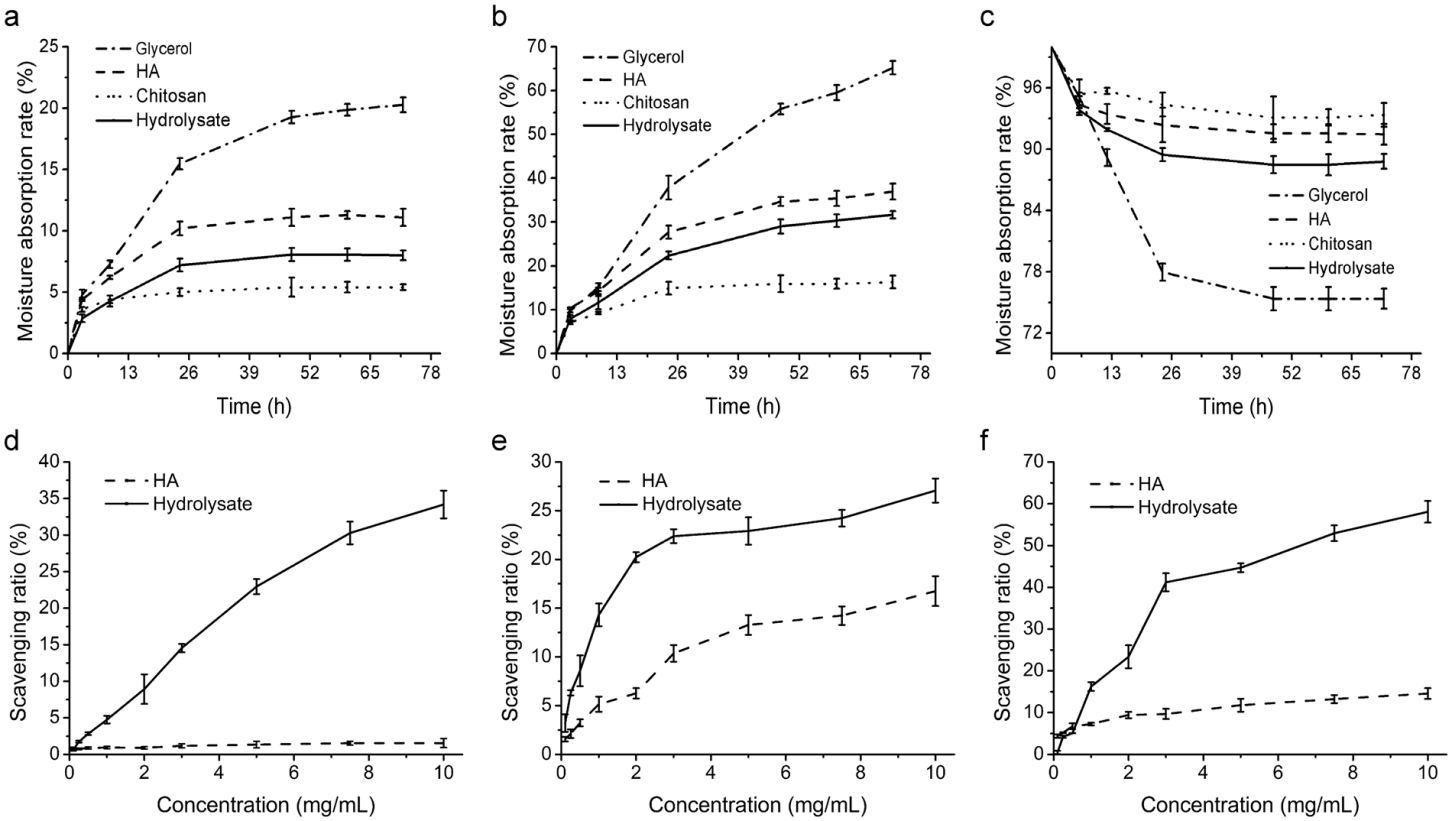
Bioactivity analyses of the plant-scale hydrolysate. (**a**) Moisture-absorption abilities in a saturated K_2_CO_3_ desiccator (43% RH) at 25 °C. (**b**) Moisture-absorption abilities in a saturated (NH_4_)_2_SO_4_ desiccator (81% RH) at 25 °C. (**c**) Moisture-retention ability in a silica gel desiccator at 25 °C. (d) DPPH∙ scavenging activity. (**e**) ∙OH scavenging activity. (**f**) O_2_^−^∙ scavenging activity.

While collagen peptides are well recognized as an ideal material in cosmetic industry^31^, fish skin collagen peptides still draw little attention. Here, we investigated the moisture-absorption (*R*_*a*_) and retention abilities (*R*_*h*_) of the hydrolysate we prepared at plant level. Hyaluronic acid (HA), chitosan and glycerol, which are commonly regarded as humectant agents in cosmetics and industry^32^, were used as positive controls. As shown in Fig. 6a, after 72 h at 43% relative humidity (RH), the rank for the *R*_*a*_ of all tested samples was as follows: glycerol > HA > fish skin hydrolysate > chitosan. At 81% RH, the ranking for the *R*_*a*_ of all tested samples was the s_*a*_me as that at 43% RH (Fig. 6b). This result indicated that the moisture-absorption rate of the hydrolysate was higher than that of chitosan, reaching 8 ± 0.52% at 43% RH and 31 ± 0.72% at 81% RH, respectively. In addition, the *R*_*h*_ of the hydrolysate was superior to glycerol, reaching 88 ± 0.72% after the hydrolysate was dehydrated for 72 h in a silica gel chamber (Fig. 6c).

### Antioxidant activity of the hydrolysate

The antioxidant activity of the plant-scale hydrolysate was assessed based on its free radical-scavenging activity. As shown in Fig. 6, the activity of the hydrolysate to scavenge free radicals increased in a concentration-dependent manner. The scavenging ratios for DPPH•, •OH and O_2_^−^• of the hydrolysate at a concentration of 10.0 mg/mL were 34 ± 1.91% (Fig. 6d), 27 ± 2.23% (Fig. 6e), and 62 ± 3.12% (Fig. 6f), respectively, much higher than those of HA, an ideal material in cosmetics with free radical-scavenging ability^31^. These data indicate that the hydrolysate has good antioxidant activity.

### Effect of the hydrolysate on the cell viability of human dermal fibroblasts

**Figure 7:**
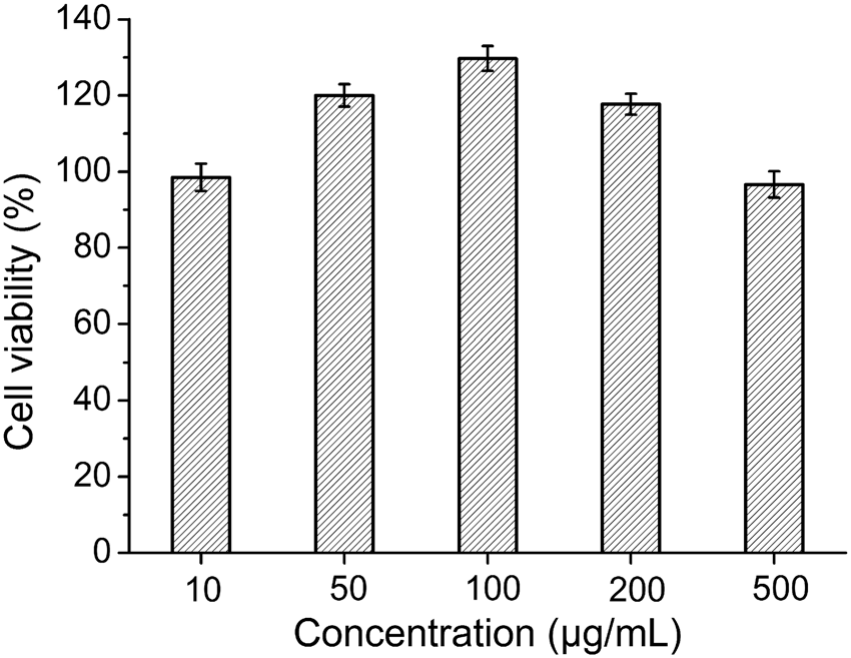
Effect of the plant-scale hydrolysate on the cell viability of human dermal fibroblasts. The fibroblasts were inoculated in a 96-well tissue culture plate with 6 × 10^3^ cells per well in DMEM containing 10% FBS and different concentrations of the hydrolysate, and were incubated at 37 °C with 5% CO_2_ for 24 h. MTT assay was performed after 24 h incubation.

Human dermal fibroblasts (HDF) were used to investigate whether the plant-scale hydrolysate has promoting effect on cell proliferation and cell viability of human skin. HDF were cultured for 24 h in Dulbecco’s modified Eagle’s medium (DMEM) containing different concentrations of the hydrolysate. As shown in Fig. 7, the cell viability of HDF increased with the increase of hydrolysate concentration, reaching the maximum (131 ± 4.43%) at the hydrolysate concentration of 100 μg/mL. This result suggests that the hydrolysate may have the function of promoting cell proliferation of human skin, which is consistent with the finding that collagen-derived dipeptides promote cell proliferation, the synthesis of hyaluronic acid and collagen deposition in human derms^33^.

### Safety assessment of the hydrolysate

**Figure 8:**
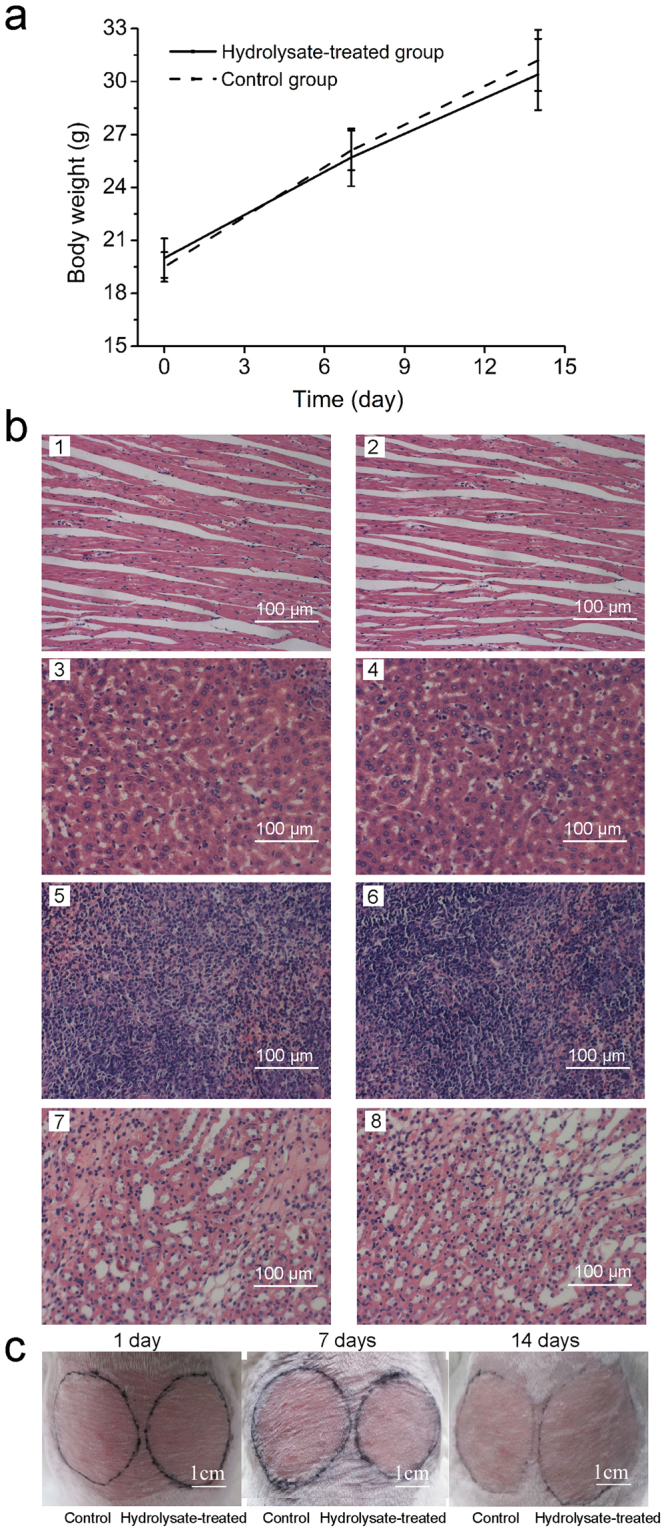
Safety assessment of the plant-scale hydrolysate. (**a**) Effect of the hydrolysate on the weight of KM mice. (**b**) Effect of the hydrolysate on the organs of KM mice. 1 and 2 are heart sections (200×); 3 and 4 are liver sections (200×); 5 and 6 are spleen sections (200×); 7 and 8 are kidney sections (200×). 1, 3, 5 and 7 are from the control group treated with H_2_O, and 2, 4, 6 and 8 are from the hydrolysate-treated group. The paraffin tissue sections were stained with hematoxylin-eosin (HE) and then observed by light microscopy. (**c**) Effect of the hydrolysate on Japan white rabbits skin in the cumulative cutaneous irritation test. Images were captured on the 0th, 7th and 14th days.

An acute toxicity test on KunMing (KM) mice and a skin irritation test on rabbits were carried out routinely to determine the bio-safety of the plant-scale hydrolysate. In the acute toxicity test, no mice died or had signs of toxicity, such as depression, hair loss, dyspnea, wound formation or any other toxicological effects in the hydrolysate-treated group (5,000 mg/kg) or the control group throughout the 14-day study. In addition, neither significant difference in the mean body weight (Fig. 8a) nor gross pathological abnormalities (such as cell accumulation or degeneration) in heart, liver, spleen or kidney were observed between the control and the treated groups (Fig. 8b). Hence, it could be concluded that the hydrolysate is safe for oral administration.

In the skin irritation test, primary cutaneous irritation (PII) and cumulative cutaneous irritation (CII) was performed with the hydrolysate at a concentration of 200 mg/mL. In the primary cutaneous irritation test, no edema, erythema, rough or thinning skin was observed in either the control or the treated group after 1, 24, 48 or 72 h (Table S3). Thus, the total score was zero and the PII of the hydrolysate was zero. In the cumulative cutaneous irritation test, no clinical signs of erythema or edema were observed on the skin treated with the hydrolysate for 14 days (Fig. 8c and Table S4). Thus the total score of the 14-day test was zero and the CII of the hydrolysate was zero. According to the criteria listed in Table S2, it could be concluded that the hydrolysate has no stimulus to skin because the PII and CII were examined as no irritation (Tables S3 and S4).

In summary, with SM9913 extracellular proteases that mainly contain a serine collagenolytic protease, a laboratory-scale process to prepare collagen oligopeptide hydrolysate from codfish skin was developed and was further scaled up to pilot and plant levels. The hydrolysate contains ~95% peptides with molecular weights lower than 3000 Da and approximately 60% lower than 1000 Da, in which collagen peptides account for approximately 95%. The hydrolysate has moisture-retention ability, antioxidant activity, and promoting effect on human skin cell proliferation, but no toxicity or skin-irritation. These results indicate that the serine collagenolytic protease of SM9913 is a good tool for preparing bioactive collagen oligopeptides from fish skin.

## Methods

### Materials and strains

Skin of codfish (*Gadous macrocephaius*) was provided by Chinese Dayudao Group (Yantai, China) and was stored at −20 °C in polyethylene bags before hydrolysis. Strain SM9913, previously isolated from 1,855 m deep-sea sediment^23^, was routinely cultured at 20 °C for 24 h in a sea water liquid medium (pH 8.0) containing 10 g/L peptone (Oxoid, England) and 5 g/L yeast extract (Oxoid, England), and then stored at 4 °C for short-term use. Dimethyl sulphoxide (DMSO), 1,1-diphenyl-2-picryl-hydrazylradical (DPPH•) and 3-(4,5-dimethylththiazoyl-2-yl)−2,5-diphenylte-2-H-trazoliumbromide (MTT) were purchased from Sigma (USA). DMEM and fetal bovine serum (FBS) were purchased from Gibco (USA). Human dermal fibroblasts, Japan white rabbits and KM mice were purchased from Shandong Academy of Medical Sciences, China. Insoluble type I collagen fiber (bovine Achilles tendon) was purchased from Worthington Biochemical Corporation (USA). All chemicals used in this study were of analytical reagent grade. Wheat bran, corn powder and bean powder were bought from a local farmer market.

### Fermentation optimization and pilot-scale production of the extracellular protease of SM9913

As previously described^23^, the medium for extracellular protease production of SM9913 was composed of (w/v) 2.0% corn powder, 2.0% bean powder, 1.0% wheat bran, 1.0% Na_2_HPO_4_, 0.03% KH_2_PO_4_, 0.1% CaCl_2_ and artificial sea water with an initial pH of 8.0. Small-scale fermentation in a 3 L fermentor (Bioflo/Celligen 310, New Brunswick Scientific, USA) was conducted at 15 °C for 84 h with a loading volume of 2.1 L and 1% (v/v) inoculum, and two key factors, gas flow and stirring speed, were optimized for protease production. Pilot-scale fermentation was conducted at 15 °C for 84 h in a 200 L fermentor (GHJ9-100, Shanghai Guoqiang Biochemical Engineering Equipment Co., Ltd., China) with a loading volume of 140 L and 1% (v/v) inoculum. Fermentation aeration rate and stirring speed were optimized according to the protease production during pilot-scale fermentation. The fermented broth was centrifuged at 12,000 rpm at 4 °C for 20 min, and the supernatant was collected for further use in fish skin hydrolysis. Protease activity towards casein was measured as previously described^23^. All treatments were repeated three times.

### Optimization of enzymatic hydrolysis parameters by single factor experiments

In the process of enzymatic hydrolysis of fish skin, three parameters, E/S, hydrolysis temperature and hydrolysis time, were optimized by single factor experiments. In brief, the reaction mixture containing 10 g of minced fish skin and 20 mL enzyme solution with different enzyme-substrate ratio (0, 4,000 U/kg, 6,000 U/kg, 12,000 U/kg, 16,000 U/kg or 26,000 U/kg) was put in a 250 mL Erlenmeyer flask. Enzymatic hydrolysis was performed at pH 8.0 at different hydrolysis temperature (20 °C, 37 °C, 45 °C, 50 °C or 55 °C) for different hydrolysis time (0.5 h, 1.0 h, 1.5 h or 2.0 h) in a thermostatically controlled water-bath with constant agitation (150 rpm). Hydrolysis reaction was terminated by heating the reaction mixture at 90 °C for 15 min, and the resulting slurry was centrifuged and the residual solid fish skin was weighed. For each parameter, the response was the amount of the residual solid skin substrate.

### Production of fish skin hydrolysate on a laboratory scale and its scale-up to pilot and plant levels

For the laboratory scale, the reaction mixture was composed of 10 g minced fish skin and 20 mL crude enzyme solution (E/S at 20,000 U/kg) in a 250 mL Erlenmeyer flask. Enzymatic hydrolysis was carried out at pH 8.0 and 40 °C for 1.5 h in a thermostatically controlled water-bath with constant agitation (150 rpm). Then, the reaction was terminated with a 15-min incubation at 90 °C. After centrifugation at 12,000 rpm, 4 °C for 15 min, supernatant was collected, freeze dried and stored at 4 °C for further analysis. For pilot scale (100 L thermostatically stirred batch reactor) and plant scale (2,000 L thermostatically stirred batch reactor), fish skin was homogenized and emulsified in distilled water at a ratio of 1:1 (w/w) by continuous stirring and then enzyme solution was added at a ratio of 20,000 U/kg. All digestions were carried out at pH 8.0 and 40 °C with stirring (150 rpm) for 4 h, and then the reaction mixture was heated to 90 °C for 15 min. The hydrolysis mixture was filtered by plate-frame pressure filtration and the resulting supernatant was condensed with a vacuum concentration tanker (RD500, GuanYi Mechanical Equipment Co., Ltd, China) and dried by a spray dryer (BoDaCo., China) at a 10 kg/h flow rate with a 170 °C inlet temperature and a 90 °C outlet temperature.

### Characterization of the hydrolysates from different levels

Peptides and amino acids in the hydrolysates from different levels were analyzed using the methods previously described^34^. Briefly, free amino acids and total amino acids in the hydrolysates were analyzed on HITACHI 835, respectively. The amount of peptides was determined by subtracting the amount of free amino acids from that of total amino acids. RP-HPLC (Waters alliance 2695, USA) coupled with a dual wavelength UV detector 2487 and a symmetry C18 column (250 mm × 4.6 mm) (Waters, Milford, MA, USA) was used to analyze the peptide spectra of the hydrolysates^35^. The sample was prepared in 50 mg/mL with deionized water. MALDI-TOF-MS (Bruker Daltonics, Bremen, Germany) was used to investigate the molecular masses of the peptides in the hydrolysates. Size exclusion chromatography was used to analyze the molecular mass distribution of peptides in the hydrolysates with the menthod described by Gu *et al*.^36^. Briefly, the molecular mass distributions for the hydrolysates from fish skin were analyzed by gel permeation chromatography on a TSK gel G2000 SWXL column (300 × 7.8 mm; Tosoh, Tokyo, Japan) using a LC-20A high performance liquid chromatography (HPLC) system (Shmadzu, Kyoto, Japan). The mobile phase used was acetonitrile/water (45:55, v/v) in the presence of 0.1% (v/v) trifluoroacetic acid. Samples (2.0 mg/mL) were eluted at a flow rate of 0.5 mL/min and monitored at 220 nm at 30 °C. The calibration standards for molecular mass contain tripeptide GGG (Mr 189) and tetrapeptide GGYR (Mr 451) from China Peptides Co. Ltd (Shanghai, China), and bacitracin (Mr 1422), aprotinin (Mr 6511) and cytochrome C (Mr 12400) from Sigma Chemical Co. (St. Louis, USA). The chromatogram was sorted into several fractions based on the molecular weight (<1000 Da, 1000–3000 Da, 3000–10000 Da and >10000 Da) by manually setting the starting and ending points of the fractions. The content of each fraction was calculated based on the relative area of each fraction in percent of the total area of all fractions.

### Collagen extraction and characterization

Collagen was extracted from codfish skin according to the method described by Song *et al*. with minor modification^37^. Briefly, codfish skins were washed thoroughly, cut into small pieces and extracted twice with 0.5 M acetic acid for 5 d. After centrifugation, NaCl powder was added to the supernatant to a final concentration of 2.6 M. The precipitated collagen was dissolved in 0.5 M acetic acid, dialyzed against distilled water for 24 h, and then freeze-dried. The collagen from codfish skin was assayed by SDS-PAGE using 10% resolving gel and 5% stacking gel with type I collagen from bovine Achilles tendon as a standard.

Amino acid composition of the collagen and the codfish skin were analyzed on HITACHI 835. The molecular masses of the peptides released from the collagen by the extracellular protease from SM9913 after digestion at 60 °C for 5 h was analyzed by MALDI-TOF-MS as described above.

### Moisture-absorption and retention abilities and antioxidant activity of the hydrolysate

The moisture-absorption and retention abilities of the plant-scale hydrolysate and control samples were investigated with the method described by Sun *et al*. (2015)^38^. Control samples included hyaluronic acid (HA), chitosan and glycerol.

To determine the antioxidant activity, the capacities of the plant-scaled hydrolysate for scavenging hydroxyl radical (•OH), DPPH• and superoxide anion (O_2_^−^•) were measured with the method described by Sun *et al*.^38^. HA was used as a positive control due to its capability of scavenging free radicals^39^.

### Effect of the hydrolysate on the proliferation of human dermal fibroblasts

MTT test was adopted to evaluate the effect of the plant-scale hydrolysate on the proliferation of HDF by the method previously described^40^ with slight modifications. The fibroblasts were cultured in DMEM with 10% FBS for 2 days at 37 °C with 5% CO_2_ and maintained to 80–90% confluence. Then the cells were suspended and inoculated into a 96-well tissue culture plate with 6 × 10^3^ cells/well in DMEM supplemented with 10% FBS and the hydrolysate solution at different concentrations (10, 50, 100, 200 or 500 μg/mL) for 24 h.

### Safety assessment of the fish skin hydrolysate

An acute toxicity test on 20 female KM mice (6–8 weeks old, 19–21 g) and a skin irritation test on 10 Japan white rabbits (1.5–1.7 kg, five males and five females) were strictly performed following the guidelines of the Good Laboratory Practice Standards Manual and the Organization for Economic Co-operation and Development (OECD). The Guideline 420 (No, 2001)^41^ was adopted for the acute oral toxicity study and the Guideline 404 (OECD, 2002)^42^ for the skin irritation study. In the acute toxicity test, the treated group was administrated with a single dose of 5,000 mg/kg of the hydrolysate. In the skin irritation test, the usage of the hydrolysate was 0.5 mL (200 mg/mL). Scores of clinical signs of skin irritation were given after 1, 24, 48 and 72 h based on the criteria listed in Table S1. The primary cutaneous irritation index (PII) was the average score calculated as follows: total grades of erythema or edema for all rabbits in a group were divided by the number of animals. For the cumulative cutaneous irritation testing, the above treatment once daily was repeated for 14 days. Meanwhile, grades of signs of erythema or edema were given daily 1 h after the hydrolysate solution was wiped. The cumulative cutaneous irritation index (CII) was the average score calculated as follows: sum of edema and erythema grade for all rabbits was divided by the number of rabbits in the group and testing days. Subsequently, the irritation intensity of the hydrolysate was determined according to Table S2. The present study was approved by the Center for New Drugs Evaluation of Shandong University’s review committee and Shandong University’s Animal Ethics Committee and performed in accordance with the Guidelines for Laboratory Animal Use and Care from the Chinese Center for Disease Control and Prevention and the Rules for Medical Laboratory Animals (1998) from the Chinese Ministry of Health.

## Electronic supplementary material

Supplementary Information

## References

[1] Raghavan S, Kristinsson HG (2009). ACE-inhibitory activity of tilapia protein hydrolysates. Food Chem..

[2] Salampessy J (2010). Release of antimicrobial peptides through bromelain hydrolysis of leatherjacket (*Meuchenia* sp.) insoluble proteins. Food Chem..

[3] Samaranayaka AG, Li-Chan EC (2008). Autolysis-assisted production of fish protein hydrolysates with antioxidant properties from Pacific hake (*Merluccius productus*). Food chem..

[4] Yang R (2009). Immunomodulatory effects of marine oligopeptide preparation from chum salmon (*Oncorhynchus keta*) in mice. Food Chem..

[5] Chalamaiah M, Rao GN, Rao D, Jyothirmayi T (2010). Protein hydrolysates from meriga (*Cirrhinus mrigala*) egg and evaluation of their functional properties. Food Chem..

[6] Adler-Nissen, J. Enzymic hydrolysis of food proteins. Elsevier Applied Science Publishers: London, UK, pp. 122–124 (1986).

[7] Benito-Ruiz P (2009). A randomized controlled trial on the efficacy and safety of a food ingredient, collagen hydrolysate, for improving joint comfort. Int. J. Food Sci. Nutr..

[8] Rajanbabu V, Chen JY (2011). Applications of antimicrobial peptides from fish and perspectives for the future. Peptides.

[9] Zhou SL, Wang HY, Yue DX (2011). Clinical effects and satefy of oral treatment with low-molecular fish collagen hydrolysate on female facial skin properties. J. Pract. Dermatol..

[10] Najafian L, Babji AS (2012). A review of fish-derived antioxidant and antimicrobial peptides: their production, assessment, and applications. Peptides.

[11] Marchbank T, Limdi JK, Mahmood A, Elia G, Playford RJ (2008). Clinical trial: protective effect of a commercial fish protein hydrolysate against indomethacin (NSAID)-induced small intestinal injury. Aliment. Pharmacol. Ther..

[12] Nesse KO, Nagalakshmi AP, Marimuthu P, Singh M (2011). Efficacy of a fish protein hydrolysate in malnourished children. Indian J. Clin. Biochem..

[13] Guérard F (2010). Recent developments of marine ingredients for food and nutraceutical applications: a review. J. Sci. Hal. Aquat..

[14] Asserin J, Lati E, Shioya T, Prawitt J (2015). The effect of oral collagen peptide supplementation on skin moisture and the dermal collagen network: evidence from an *ex vivo* model and randomized, placebo-controlled clinical trials. J. Cosmet. Dermatol..

[15] Khantaphant S, Benjakul S (2008). Comparative study on the proteases from fish pyloric caeca and the use for production of gelatin hydrolysate with antioxidative activity. Comp. Biochem. Physiol. B Biochem. Mol. Biol..

[16] Phanturat P, Benjakul S, Visessanguan W, Roytrakul S (2010). Use of pyloric caeca extract from bigeye snapper (*Priacanthus macracanthus*) for the production of gelatin hydrolysate with antioxidative activity. LWT-Food Sci. Technol..

[17] Okamoto M, Yonejima Y, Tsujimoto Y, Suzuki Y, Watanabe K (2001). A thermostable collagenolytic protease with a very large molecular mass produced by thermophilic *Bacillus* sp. strain MO-1. Appl. Microbiol. Biotechnol..

[18] Zhao GY (2008). Hydrolysis of insoluble collagen by deseasin MCP-01 from deep-sea *Pseudoalteromonas* sp. SM9913: collagenolytic characters, collagen-binding ability of C-terminal polycystic kidney disease domain, and implication for its novel role in deep-sea sedimentary particulate organic nitrogen degradation. J. Biol. Chem..

[19] Huo JX, Zheng Z (2009). Study on enzymatic hydrolysis of *Gadus morrhua* skin collagen and molecular weight distribution of hydrolysates. Agr. Sci. China.

[20] Gu RZ, Li CY, Liu WY, Yi WX, Cai MY (2011). Angiotensin I-converting enzyme inhibitory activity of low-molecular-weight peptides from Atlantic salmon (Salmo salar L.) skin. Food Res. Int..

[21] Yang Y, Yan H, Ding G, Huang F (2011). Isolation and purification of an anticancer activity peptide from protein hydrolysate of *Mytilus coruscus*. J. China Pharm. Univ..

[22] Pei X (2010). Marine collagen peptide isolated from Chum Salmon (*Oncorhynchus keta*) skin facilitates learning and memory in aged C57BL/6J mice. Food chem..

[23] Chen XL, Zhang YZ, Gao PJ, Luan XW (2003). Two different proteases produced by a deep-sea psychrotrophic bacterial strain, *Pseudoaltermonas* sp. SM9913. Mar. Biol..

[24] Shao X (2015). Culture condition optimization and pilot scale production of the M12 metalloprotease myroilysin produced by the deep-sea bacterium *Myroides profundi* D25. Molecules.

[25] Ran LY (2013). Structural and mechanistic insights into collagen degradation by a bacterial collagenolytic serine protease in the subtilisin family. Mol. Microbiol..

[26] Gao X (2010). Structural basis for the autoprocessing of zinc metalloproteases in the thermolysin family. Proc. Natl. Acad. Sci. USA.

[27] Chalamaiah M, Hemalatha R, Jyothirmayi T (2012). Fish protein hydrolysates: proximate composition, amino acid composition, antioxidant activities and applications: a review. Food Chem..

[28] Bae I (2008). Biochemical properties of acid-soluble collagens extracted from the skins of underutilised fishes. Food chem..

[29] Osatomi K (2016). Rapid isolation of high purity pepsin-soluble type I collagen from scales of red drum fish (*Sciaenops ocellatus*). Food Hydrocoll..

[30] Chen S (2016). Isolation, characterization and valorizable applications of fish scale collagen in food and agriculture industries. Biocatal Agric Biotech..

[31] Wang L (2008). Isolation and characterisation of collagens from the skin, scale and bone of deep-sea redfish (*Sebastes mentella*). Food Chem..

[32] Sun L (2006). Conversion of crystal structure of the chitin to facilitate preparation of a 6-carboxychitin with moisture absorption–retention abilities. Carbohydr. Polym..

[33] Ohara H (2010). Collagen-derived dipeptide, proline-hydroxyproline, stimulates cell proliferation and hyaluronic acid synthesis in cultured human dermal fibroblasts. J. Dermatol..

[34] He H, Chen X, Sun C, Zhang Y, Gao P (2006). Preparation and functional evaluation of oligopeptide-enriched hydrolysate from shrimp (*Acetes chinensis*) treated with crude protease from *Bacillus* sp. SM98011. Bioresour. Technol..

[35] Wang YK (2010). Oyster (*Crassostrea gigas*) hydrolysates produced on a plant scale have antitumor activity and immunostimulating effects in BALB/c mice. Mar. Drugs.

[36] Gu RZ (2012). Antioxidant and angiotensin I-converting enzyme inhibitory properties of oligopeptides derived from black-bone silky fowl (Gallus gallus domesticus Brisson) muscle. Food Res. Int..

[37] Song E (2006). Collagen scaffolds derived from a marine source and their biocompatibility. Biomaterials.

[38] Sun ML (2015). Characterization and biotechnological potential analysis of a new exopolysaccharide from the Arctic marine bacterium *Polaribacter* sp. SM1127. Sci. Rep..

[39] Sato H (1988). Antioxidant activity of synovial fluid, hyaluronic acid, and two subcomponents of hyaluronic acid. Synovial fluid scavenging effect is enhanced in rheumatoid arthritis patients. Arthritis Rheum..

[40] You DH, Nam MJ (2013). Effects of human epidermal growth factor gene-transfected mesenchymal stem cells on fibroblast migration and proliferation. Cell Prolif..

[41] OECD. OECD guidelines for acute toxicity of chemicals, no. 420. *Organisation for Economic Co-operation and development*, *Paris, France* (2001).

[42] OECD. OECD guidelines for acute dermal irritation/corrosion of chemicals, no. 404. *Organization for economic cooperation and development, Paris, France* (2002).

