# Supplementary material for: Preparation and functional evaluation of collagen oligopeptide-rich hydrolysate from fish skin with the serine collagenolytic protease from Pseudoalteromonas sp. SM9913
Source: Sci Rep. 2017 Nov 16;7:15716. doi: 10.1038/s41598-017-15971-9 (PMC5691207; doi:10.1038/s41598-017-15971-9)
Supplement: Supplementary file 1 — Supplementary Information [file 41598_2017_15971_MOESM1_ESM.doc]

**Supplementary information**

**Preparation and functional evaluation of** **collagen oligopeptide-rich hydrolysate from fish skin with** **the serine collagenolytic protease from *Pseudoalteromonas* sp. SM9913**

Xiu-Lan Chen^1,$^, Ming Peng^1, $^, Jing Li^1,$^, Bai-Lu Tang^1^, Xuan Shao^1^, Fang Zhao^1^, Chang Liu^1^, Xi-Ying Zhang^1^, Ping-Yi Li, Mei Shi^1^, Yu-Zhong Zhang^1,2^, Xiao-Yan Song^1,2,#^

^1^State Key Laboratory of Microbial Technology, Marine Biotechnology Research Center, Institute of Marine Science and Technology, Shandong University, Jinan 250100, China

^2^Laboratory for Marine Biology and Biotechnology, Qingdao National Laboratory for Marine Science and Technology, Qingdao 266237, China

$ Xiu-Lan Chen, Ming Peng and Jing Li contributed equally to this work.

# Corresponding author. E-Mail: xysong@sdu.edu.cn; Tel: +86-531-88365013; Fax: +86-531-88564326.

The following tables and Figure are included as Supplementary Information

**Supplementary Table S1.** Irritant effects of the plant-scale hydrolysate on Japan white rabbit skin in primary cutaneous irritation test.

**Supplementary Table S2.** Irritant effects of the plant-scale hydrolysate on Japan white rabbit skin in cumulative cutaneous irritation test.

**Supplementary Table S3.** Grading standard for skin irritation based on OECD guidelines.

**Supplementary Table S4.** Evaluation standard for skin irritation intensity based on OECD guidelines.

**Table S1.** Irritant effects of the plant-scale hydrolysate on Japan white rabbit skin in primary cutaneous irritation test.

| Animal  number |  | Average score | | | | | | | | Irritant  intensity |
| --- | --- | --- | --- | --- | --- | --- | --- | --- | --- | --- |
|  |  | 1 h | | 24 h | | 48 h | | 72 h | |  |
|  | Sex | Hydrolysate-treated skin | Control skin | Hydrolysate-treated skin | Control skin | Hydrolysate-treated skin | Control skin | Hydrolysate-treated skin | Control skin |  |
| 1 | Male | 0 | 0 | 0 | 0 | 0 | 0 | 0 | 0 | NO |
| 2 | Male | 0 | 0 | 0 | 0 | 0 | 0 | 0 | 0 | NO |
| 3 | Male | 0 | 0 | 0 | 0 | 0 | 0 | 0 | 0 | NO |
| 4 | Male | 0 | 0 | 0 | 0 | 0 | 0 | 0 | 0 | NO |
| 5 | Male | 0 | 0 | 0 | 0 | 0 | 0 | 0 | 0 | NO |
| 6 | Female | 0 | 0 | 0 | 0 | 0 | 0 | 0 | 0 | NO |
| 7 | Female | 0 | 0 | 0 | 0 | 0 | 0 | 0 | 0 | NO |
| 8 | Female | 0 | 0 | 0 | 0 | 0 | 0 | 0 | 0 | NO |
| 9 | Female | 0 | 0 | 0 | 0 | 0 | 0 | 0 | 0 | NO |
| 10 | Female | 0 | 0 | 0 | 0 | 0 | 0 | 0 | 0 | NO |

**Table S2.** Irritant effects of the plant-scale hydrolysate on Japan white rabbit skin in cumulative cutaneous irritation test.

| Time  (day) | Average score | | | | | | | | | Irritant  intensity |
| --- | --- | --- | --- | --- | --- | --- | --- | --- | --- | --- |
|  | Rabbit 1 | | | Rabbit 2 | | Rabbit 3 | | Rabbits 4-10 | |  |
|  | Hydrolysate-treated skin | Control skin | Hydrolysate-treated skin | | Control skin | Hydrolysate-treated skin | Control skin | Hydrolysate-treated skin | Control skin |  |
| 1 | 0 | 0 | 0 | | 0 | 0 | 0 | 0 | 0 | NO |
| 2 | 0 | 0 | 0 | | 0 | 0 | 0 | 0 | 0 | NO |
| 3 | 0 | 0 | 0 | | 0 | 0 | 0 | 0 | 0 | NO |
| 4 | 0 | 0 | 0 | | 0 | 0 | 0 | 0 | 0 | NO |
| 5 | 0 | 0 | 0 | | 0 | 0 | 0 | 0 | 0 | NO |
| 6 | 0 | 0 | 0 | | 0 | 0 | 0 | 0 | 0 | NO |
| 7 | 0 | 0 | 0 | | 0 | 0 | 0 | 0 | 0 | NO |
| 8 | 0 | 0 | 0 | | 0 | 0 | 0 | 0 | 0 | NO |
| 9 | 0 | 0 | 0 | | 0 | 0 | 0 | 0 | 0 | NO |
| 10 | 0 | 0 | 0 | | 0 | 0 | 0 | 0 | 0 | NO |
| 11 | 0 | 0 | 0 | | 0 | 0 | 0 | 0 | 0 | NO |
| 12 | 0 | 0 | 0 | | 0 | 0 | 0 | 0 | 0 | NO |
| 13 | 0 | 0 | 0 | | 0 | 0 | 0 | 0 | 0 | NO |
| 14 | 0 | 0 | 0 | | 0 | 0 | 0 | 0 | 0 | NO |

**Table S3. Grading standard for skin irritation based on OECD guidelines.**

| Irritation response | Score |
| --- | --- |
| Erythema |  |
| No | 0 |
| Very slight (barely perceptible) | 1 |
| Well defined | 2 |
| Moderate to severe | 3 |
| Severe (beef redness) to eschar formation preventing grading of erythema | 4 |
| Edema |  |
| No | 0 |
| Very slight (barely perceptible) | 1 |
| Slight (edges of area well defined by definite raising) | 2 |
| Moderate (raised approximately 1 mm) | 3 |
| Severe (raised more than 1 mm and extending beyond area of exposure) | 4 |
| Highest total value | 8 |

**Table S4.** Evaluation standard for skin irritation intensity based on OECD guidelines.

| Score | Evaluation |
| --- | --- |
| 0–0.49 | No irritation |
| 0.5–2.99 | Slight |
| 3.0–5.99 | Moderate |
| 6.0–8.00 | Severe |
